# Factors associated with successful vaginal birth after one lower uterine transverse cesarean section delivery

**DOI:** 10.1038/s41598-023-36027-1

**Published:** 2023-05-31

**Authors:** Tigist Derebe Tesfahun, Amlaku Mulat Awoke, Mezgebu Mihiret Kefale, Wondu Feyisa Balcha, Amanuel Tebabal Nega, Tigist Wubet Gezahegn, Bezawit Abeje Alemayehu, Magarsa Lami Dabalo, Tewodros Worku Bogale, Zigijit Azene, Selamawit Nigatu, Aberash Beyene

**Affiliations:** 1 Department of Midwifery, College of Medicine and Health Sciences, Bahir Dar University, Bahir Dar, Ethiopia; 2 Department of Midwifery, College of Medicine and Health Sciences, Haramaya University, Haramaya, Ethiopia; 3 Department of Midwifery, School of Health Sciences, Injibara University, Injibara, Ethiopia; 4 Department of Midwifery, College of Medicine and Health Sciences, Wachemo University, Hosanna, Ethiopia; 5 Department of Midwifery, College of Medicine and Health Sciences, Wolkite University, Wolkite, Ethiopia

**Keywords:** Health care, Medical research, Risk factors, Signs and symptoms

## Abstract

A Trial of labor after cesarean section is an attempt to deliver vaginally by a woman who had a previous cesarean delivery and when achieved by a vaginal delivery it is called successful vaginal birth after cesarean section. Vaginal birth after a caesarian section is a preferred method to decrease complications associated with repeated caesarian section delivery for both mother and fetus. It has a higher success rate when the right women are selected for a trial of labor. This study aimed to assess factors associated with successful vaginal birth after one lower uterine transverse cesarean section and to validate the Flamm and Geiger score at the public hospitals of Bahir Dar City, Northwest, Ethiopia, 2021. A health facility-based retrospective cross-sectional study was conducted from March 1 to 15/2021. A medical record review of 408 women charts with a trial of labor after one lower uterine transverse cesarean section from January 1/2020 to December 31/2020 was done and 345 women charts with complete maternal and fetal information were included in the study with a response rate of 84.6%. The data were collected using a structured checklist, entered into Epi data 3.1, and analyzed using SPSS 25.0 version. Logistic regression analyses were done to estimate the crude and adjusted odds ratio with a confidence interval of 95% and a P-value of less than 0.05 considered statistically significant. This study identified that the trial of labor after cesarean section rate was 69.5%, and the success rate of vaginal birth after one lower uterine transverse cesarean section was 35.07%. Of the failed trial of labor, fetal distress (38.9%) and failed progress of labor (32.1%) were the main indications for an emergency cesarean section. The maternal age group of 21–30 years, prior vaginal birth after or before cesarean section, non-recurring indication (fetal distress and malpresentation), ruptured membrane, cervical dilatation ≥ 4 cm, cervical effacement ≥ 50%, and low station (≥ 0) at admission were associated with successful vaginal birth after one lower uterine transverse cesarean section. For the Flamm and Geiger score at a cut point of 5, the sensitivity and specificity were 73.6% and 86.6% respectively. In this study area, the trial of labor after cesarean section rate is encouraging, however, the success rate of vaginal birth after one lower uterine transverse caesarian section was lower. The maternal socio-demographic and obstetric-related factors were significantly associated with successful vaginal birth after one lower transverse caesarian section delivery. This study indicated that when the Flamm and Geiger score increases, the chance of successful vaginal birth after one lower uterine transverse caesarian section also increases. We suggest emphasizing counselling and encouraging the women, as their chance of successful vaginal delivery will be high in the subsequent pregnancy, especially if the indications of primary caesarian section delivery were non-recurring.

## Introduction

With the increasing trends in cesarean section (CS) rates, a large expanding population of women with CS is being confronted with various problems in their future pregnancies, particularly to their mode of delivery^1^. Women who had CS in previous births have two options for their care in a subsequent pregnancy: planned elective repeat cesarean delivery (RCD) or planned vaginal delivery after caesarian section (VBAC)^2^. Both options have inherent benefits and risks. However, there is evidence of a more favorable benefit-risk ratio for planned VBAC compared with RCD^3,4^.

Planned VBAC without contraindication is the preferred method for women who have a singleton pregnancy with cephalic presentation at 37 + 0 weeks or more and with or without a history of previous vaginal birth^4,5^. The overall proportion of successful VBAC was greater than 60%, if the primary CS was done for non-recurring indications: such as for fetal distress, poor labor progress, placenta previa, malpresentations, pregnancy-induced hypertension, and twin pregnancy^6–8^.

The reason for the increase in CS rate is multifactorial, but a recent analysis of the data concludes that the practice of elective RCD for women with a history of previous CS is the major contributor to the CS birth epidemic^6,7,9–11^. Non-medical indications also play a major role in escalating the CS rate^12,13^. Different studies conducted in Ethiopia showed that the indication of primary CS was: fetal distress, cephalo pelvic disproportion, malpresentations, previous scar (4.9–25.8%), antepartum hemorrhage, severe preeclampsia/eclampsia, and multiple pregnancies. Of these, maternal indications accounted for 2/3 and fetal indications for 1/3 of CS^14–17^.

In women who had successful VBAC, the risks associated with a trial of labor (TOL) are low. However, in women with a failed VBAC or women undergoing intrapartum emergency RCD, both risks of maternal and neonatal morbidity are increased^18–21^. RCD increases the risk of an adherent placenta, cesarean hysterectomy, uterine rupture, scar dehiscence, massive bleeding, need for blood transfusion, wound infection, endometritis, longer hospitalization, delayed initiation of breastfeeding, and increases mortality^20–24^. In Ethiopia, the maternal complications rate due to primary or secondary and above CS was 30.1%-38.2%^25,26^. About 10–25% of women who have given birth through CS developed surgical site infections^27,28^. Neonates born with RCD have an increased risk of breathing problems and increased risks of childhood obesity, asthma, and diabetes^29,30^.

Successful VBAC might reduce these risks with a shorter hospital stay, increase the chance of successful future VBACs, reduced risk of infections, reduced blood loss, reduced risk of hysterectomy, reduce bowel or bladder injury, reduce placental disorders, decrease anesthetic associated complications, confirms pelvic adequacy for a vaginal birth, decreased risk of complications in future pregnancies, has physical and psychological benefits for both the mother, and the baby, and has the advantage of early breastfeeding initiation^3,23,31–36^.

Many researchers identify factors associated with a successful VBAC such as previous vaginal birth before CS, previous VBAC, pre-pregnancy body mass index, higher bishop score, younger maternal age, spontaneous onset of labor, amniotic membrane status, cervical status at admission, fetal station, birth weight, and non-recurring indication for primary CS like fetal malpresentations^8,37–40^. Two-thirds of women with a prior lower uterine transverse cesarean section (LUTCS) are candidates for trial of labor after caesarian section (TOLAC) and should be counseled and offered this option at an institution staffed by well-trained personnel with adequate and available resources of operation^41,42^.

The right to choose the mode of delivery is a crucial component of compassionate and respectful care in modern obstetrics as it fosters both maternal and neonatal well-being^43^. Several VBAC prediction models have been developed to support the counseling process and informed decision making. Some of the models use antepartum variables collected during antenatal (ANC) visits, whereas others use both the antepartum and intrapartum variables to predict the probability of successful TOLAC at admission for labor and delivery services^44^. Flamm and Goings found that RCD and TOL are associated with equal risks, while the cost of TOL is less if the probability of successful TOL is more than 0.7^13^.

Even though different hospitals offer TOL for mothers with a prior LUTCS, there is no adequate study that shows the TOLAC rate and VBAC success rate in Ethiopia, particularly in this study area. Although, there is no reliable algorithm or nomogram that correctly identifies or accurately predicts the success rate of VBAC in our country. This study aimed to assess the proportion of successful vaginal birth after one LUTCS and its associated factors. Additionally, to validate the Flamm and Geiger score at the public hospitals of Bahir Dar City, Northwest, Ethiopia.

## Methods

### Study design and setting

This was a retrospective health facility-based cross-sectional study conducted from March 1 to 15/2021 in the public hospitals of Bahir Dar City. Bahir Dar is the capital city of Amhara regional state and it is located about 552 km away from Addis Ababa, the capital city of Ethiopia. The estimated population of the city for the year 2020/21 is about 518, 193 of which 265,156 are females^45^. In the city, there are two public specialized referral hospitals, one primary hospital, ten health centers, two non-governmental clinics, four private hospitals, and thirty-five private clinics. Among the public health facilities, an emergency CS were done in the three of the hospitals, namely: (Felege Hiwot Comprehensive referral specialized hospital, Tibebe Gion specialized hospital, and Addis Alem primary hospital) and in the two health centers (Han health center, and Bahir Dar health center). They provide different medical services to the people of Bahir Dar city and its surrounding zones. This study was conducted in the three public hospitals of the city.

### Study population

The study included randomly selected mothers who had one previous LUTCS scar and opted for TOLAC with the attempt of TOL in the public hospitals of Bahir Dar City from January 1/2020 to December 31/ 2020.

### Inclusion and exclusion criteria

In this study, women with one previous LUTCS, term and singleton pregnancy, women who came with spontaneous onset of labor or leakage of liquor, vertex presentation of fetus, having signed informed consent and allowed to undergo TOL by the managing physician as documented on the mother's chart were included, while women charts with incomplete maternal or fetal information (such as GA of the fetus, birth weight, position of the resenting part and cervical status at admission) were excluded.

### Sample size determination

The sample size was calculated using a single population proportion formula by considering the following assumptions: the proportion of successful VBAC in teaching hospital of Addis Ababa University was 69.4%^40^, Zα/2 = critical value for normal distribution at 95% confidence level, which is equal to 1.96 (Z value of alpha at = 0.05) or 5% level of significance (α = 0.05) and a 5% margin of error (d = 0.05). The sample size was 226, and it was adjusted by adding a 5% non-response rate and the sample size became 242. However, during the actual data collection period, we found 408 women charts who tried labor vaginally after one LUTCS from January 1/2020 to December 31/2020 in the public hospitals of Bahir Dar City. As a result of this, we included all women`s charts with a TOL after one LUTCS from January 1/2020 to December 31/2020 and the final sample size was 408 women`s charts.

### Sampling techniques

The selection of women with one previous CS and TOLAC for this study was supported by guidelines of the Obstetrics Management Protocol for Ethiopian Hospitals^46^. In Ethiopian, at public or private hospitals and health centers, which have functional operating room and trained health care professionals can perform either classical or transverse CS. After one CS delivery, the women mode of delivery in the next pregnancy will depend on the types of primary CS and the presence or absence of other indications for RCD. Women who have a primary classical CS, their next mode of delivery should be RCD (they are not allowed to trying of labor vaginally). However, women who have primary transverse CS, their next mode of delivery can be RCD if there is an indication of RCD or if they need RCD, otherwise they can try vaginal delivery after signing informed consent for TOLAC. Those women who allowed TOLAC can have successful VBAC or failed TOLAC and give birth by emergency RCD. Therefore, our study aimed to assess the success rate of VBAC among women who had one previous LUTCS.

**Figure 1:**
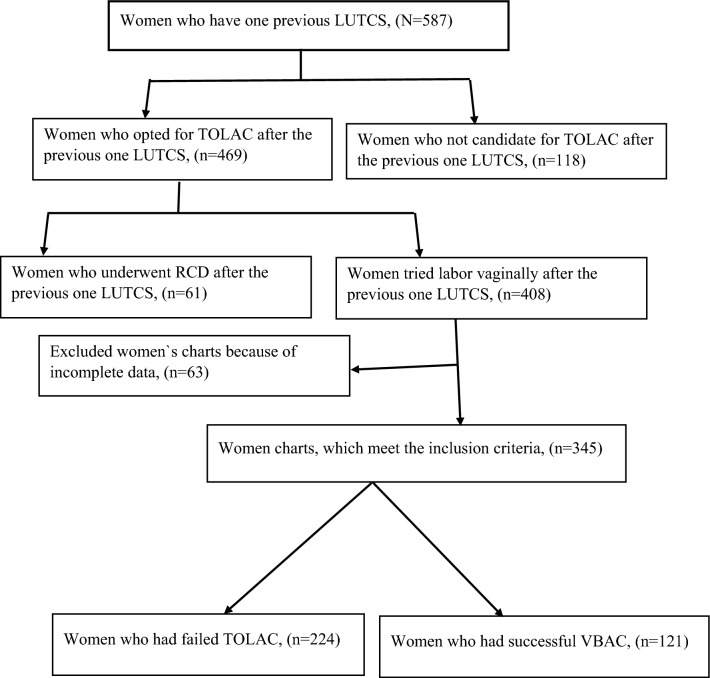
Number of women who were admitted with one previous LUTCS and sampling procedures at the public hospitals of Bahir Dar City from January 1/2020 to December 30/2020.

The primary sources of the data were the paper based admission delivery registration log books, where the card numbers of women admitted with one previous LUTCS were traced. In between January 1/2020 to December 31/2020 in Bahir Dar public hospitals, 587 women were admitted with one previous LUTCS. Then we identified women who were not candidate for TOLAC, women were underwent RCD and women who were candidate for TOLAC. Among women who were admitted with one previous LUTCS, 118 (20.1%) were not candidates for TOLAC and underwent repeat CS. The remaining, 469 (79.9%) were candidates for TOLAC, and among the candidates women 13% (61/469) were indicated a preference for RCD during admission. We found that 408 women were tried labor vaginally after previous one LUTCS with a TOLAC rate of (408/587 = 69.5%) in this study area. Then all women`s charts with only one previous LUTCS delivery, and opted for TOLAC with the attempt of TOL in each public hospital of the city from January 1/2020 to December 31/2020 were collected. Of the collected women`s chart with TOLAC after one LUTCS 63/408 (15.4%) were excluded due to incomplete data such as GA of the fetus, birth weight, position of the resenting part, cervical status at admission, and lack of informed consent. The remaining 345 women`s charts were complete, which fulfilled the inclusion criteria for TOLAC. Then the data from all fully documented charts were collected using a structured checklist (Fig. 1).

### Study variables

#### Dependent variable

Successful vaginal birth after cesarean section.

#### Independent variables

The independent variables: socio-demographic variables (maternal age and address) and obstetric and fetal factors (parity, an indication of primary CS, inter-delivery interval, prior successful VBAC, prior vaginal delivery, duration of labor, the status of the membrane at admission, and duration of rupture, cervical dilatation, and effacement at admission, fetal station, the position of the presenting part, gestational age, mode of delivery, an indication of reputed emergency CS, Timing of emergency CS and birth weight).

### Operational definitions

*Cesarean section* means delivery of the fetus, membrane, and placenta after 28 weeks of gestation by the opening of the abdomen and uterus^47^.

*Trial of labor after cesarean delivery* refers to a planned attempt to deliver vaginally by a woman who had a previous cesarean delivery^33,48^.

*Opted for TOLAC* refers to women with one previous LUTCS scar, singleton pregnancy, cephalic presentation, term pregnancy, no contraindications for vaginal delivery, documented and signed written informed consent for TOL^42^.

*Station* refer to the location of the fetal head’s lowermost portion in the pelvic canal in relation to the ischial spines, or indicate the degree of engagement of the presenting part. The stations above and below the ischial spine were categorized as the high (< 0) and low (≥ 0) in pelvic examination^40^.

*Gestational age* is calculated from the last normal menstrual period or fungal height that was documented on the card, if not from the duration of amenorrhea documented from mothers recall, and is rounded to the nearest week. Amenorrhea of 9 months was taken as 37–41^6/7^ weeks gestation for all mothers^6^.

*Elective repeat cesarean section* cesarean section done at a scheduled time for delivery before onset of labor in presence of previous CS^49^.

*Successful VBAC* a vaginal delivery (spontaneous or assisted/instrumental) in a woman who had previous one-LUTCS^33,50^.

*Failed VBAC* defined as failure to achieve a vaginal birth after a previous one-LUTCS delivery, undergoing a TOLAC, and the delivery ending by an emergency RCD^33,50^.

### Data collection tools and procedures

The data was collected using a structured checklist, which was prepared in the English language, and was adapted from relevant works of literature related to the topic^5,39,40,50^. The checklist consisted of maternal socio-demographic characteristics, past and current obstetric history, and fetal factors. A pre-tested structured checklist was used for data collection purposes. Before the actual data collection period, first, we counted the total number of women who had a previous one-LUTCS from the paper-based admission delivery registration log books and it was 587. Then we identified women who opted for TOLAC with TOL and we found 408 women who had tried labor vaginally after one previous LUTCS. After that, we collected the 408 women's charts, and the order was given based on the paper-based admission delivery registration log book numbers. Then the data were extracted using a structured checklist among women`s charts, which fulfilled the inclusion criteria until the sample size of the study was obtained. During the actual data collection period, we found 63 women's charts that had incomplete data and a lack of signed informed consent, which makes the rate of incomplete maternal or fetal information 15.4% (63/408). The data was collected by three BSc midwives and supervised by one MSc midwife.

### Data quality control

The data collectors and supervisors were trained for two days by the investigators. The checklist was pre-tested on 17 (5%) of the sample size from charts of women who had one previous LUTCS and opted for TOLAC with the attempt of TOL at Felege Hiwot comprehensive specialized Hospital in the year 2019. After necessary modifications and corrections were done to standardize and ensure its reliability and validity, additional adjustments were made based on the results of the pre-test. Based on the pretest result, we removed some variables like maternal height, weight, marital status, occupation, and variables that assess the bishop's score. Since our study was retrospective, it may not be possible to find these variables from the women's charts. The Cronbach alpha score for the pretest was 0.75. During the data collection period, daily supervision was done for data completeness.

### Data processing, and analysis

The data were entered into Epi data 3.1, edited and cleaned for inconsistencies, missing values, and outliers, then exported to SPSS version 25.0 for analysis. During the analysis, all explanatory variables which have a significant association in bivariate analysis with a P-value < 0.25 were entered into a multivariate logistic regression model to get the AOR, and those variables with 95% of CI and a P-value of < 0.05 was considered as statistically significant with successful VBAC. The multicollinearity test was done using variance inflation factor and there was no collinearity between the independent variables. The model goodness of the test was checked using the Hosmer–Lemeshow goodness of the fit and its P value was 0.637. To predict a successful TOLAC, we used the Flamm and Geiger Scoring System, which provides reasonable predictability for VBAC and also a consistent ability to identify women at risk for failed TOL. The parameter used based on Flamm and Geiger scoring system were: (1) maternal age (< 40 years = 2, above 40 years = 0), (2) vaginal birth history (before and after CS = 4, after first CS = 2, before first CS = 1, no history of vaginal birth = 0), (3) reason for first CS (failure to progress = 0, other reason = 1), (4) cervical effacement on admission (> 75% = 2, 25–75% = 1, < 25% = 0) and (5) cervical dilatation on admission (> 4 cm = 1, ≤ 4 = 0). The receiver operating characteristic (ROC) curve was measured by calculating the corresponding area under the curve (AUC). Frequency tables, figures, and descriptive summaries were used to describe the study variables.

### Ethical approval

Ethical clearance was obtained from the Institutional Review Board of Bahir Dar University, College of Medicine and Health Sciences on March 29, 2021, with IRB protocol number 211/2021. Permission to review charts was taken from each public hospital medical director and concerned bodies. The purpose of the study was explained to each public hospital medical director. The study was conducted according to the recommendations of the Declaration of Helsinki. Confidentiality was kept using anonymous codes and assured that the data would not have any negative consequences on the participants.

### Consent to participate

Informed written consent was obtained from each hospital medical director before data collection.

## Results

### Socio-demographic characteristics and obstetric factors

The total number of women who were admitted to public hospitals of Bahir Dar City with one previous LUTCS from January 1/2020 to December 31/2020 was 587, and among them 69.5% (408/587) were tried labor vaginally after one LUTCS. Of these, 345 fulfil the inclusion criteria and are included in this study, making a response rate of 84.6% (345/408). Nearly half, 170 (49.3%) of the mothers were found in the age group of 26–30 years and the women’s age ranged from 21 to 42 years. The mean age of the mothers was 28.62 years, with a standard deviation of ± 4.77. About 63% (n = 220) of the mothers live in urban areas, and 280 (81.2%) gave birth in the 25–59 months (optimal) inter-delivery interval. Of the mothers, 143 (41.4% were primipara, and failure to progress (n = 84, 24.3%) was the main indication for primary CS. Nearly one-third (29.9%) of the mothers had a history of prior spontaneous vaginal delivery and 55 (15.9%) had prior successful VBAC. Three fourth (n = 257) of the gestational age of the fetus were between 37 and 396/7 weeks with an average gestational age of 391/7 weeks and 133 (38.6%) were admitted with cervical dilatation of ≥ 4 cm. On admission, in 280 (81.2%) of the mothers the cervical effacement was ≥ 50%, and in 114 (33.0%) of the mothers, the station of the fetal head was low (≤ 0). In 94 (27.2%) of the mothers on admission, the fetal membrane was ruptured. More than half, 197 (57.1%) of mothers gave birth within 8 h, with a mean of 9.4 ± 2.7 h for successful VBAC and 6.7 ± 2.9 h for failed TOLAC, and 286 (82.9%) of the neonate birth weight were between 2500 to 4000 g (Table 1).

**Table 1:** Socio-demographic characteristics and obstetrics history of the mothers who had TOLAC in the public hospitals at Bahir Dar City, Northwest, Ethiopia, 2021, (n = 345).

Variables	No. (%)
Maternal age (years)	
21–25	87 (25.2)
26–30	170 (49.3)
31–35	58 (16.8)
36–42	30 (8.7)
Address	
Rural	125 (36.2)
Urban	220 (63.8)
Parity	
I	143 (41.4)
II	121 (35.1)
≥III	81 (23.5)
Inter delivery interval (months)	
< 24	23 (6.6)
25–59	280 (81.2)
> 60	42 (12.2)
Indication of primary CS	
Failure to progress	84 (24.3)
NRFHRP	57 (16.5)
APH	44 (12.8)
Macrosomia	24 (7.0)
Malpresentation	69 (20.0)
Unknown	22 (6.4)
Others*	45 (13.0)
Prior SVD	
Yes	103 (29.9)
No	242 (70.1)
Prior successful VBAC	
Yes	55 (15.9)
No	290 (84.1)
Gestational age (weeks)	
37–39^6/7^	257 (74.5)
40–41^6/7^	88 (25.5)
Cervical dilatation on admission	
< 4 cm	212 (61.4)
≥4 cm	133 (38.6)
Cervical effacement on admission	
< 50%	65 (18.8)
≥50%	280 (81.2)
Fetal station	
High (< 0)	231 (67.0)
Low (≥ 0)	114 (33.0)
Position of presenting part	
OA	231 (75.7)
OP/OT	84 (24.3)
Status of the membrane at admission	
Intact	251 (72.8)
Ruptured	94 (27.2)
Duration of rupture of membrane (n = 94)	
< 8 h	75 (79.8)
> 8 h	19 (20.2)
Duration of labor (h)	
< 8	197 (57.1)
≥8	148 (42.9)
Birth weight (g)	
< 2500	32 (9.3)
2500–4000	286 (82.9)
≥ 4000	27 (7.8)

*Sever preeclampsia/eclampsia, IUGR, and oligohydramnios.

### The proportion of successful VBAC

**Figure 2:**
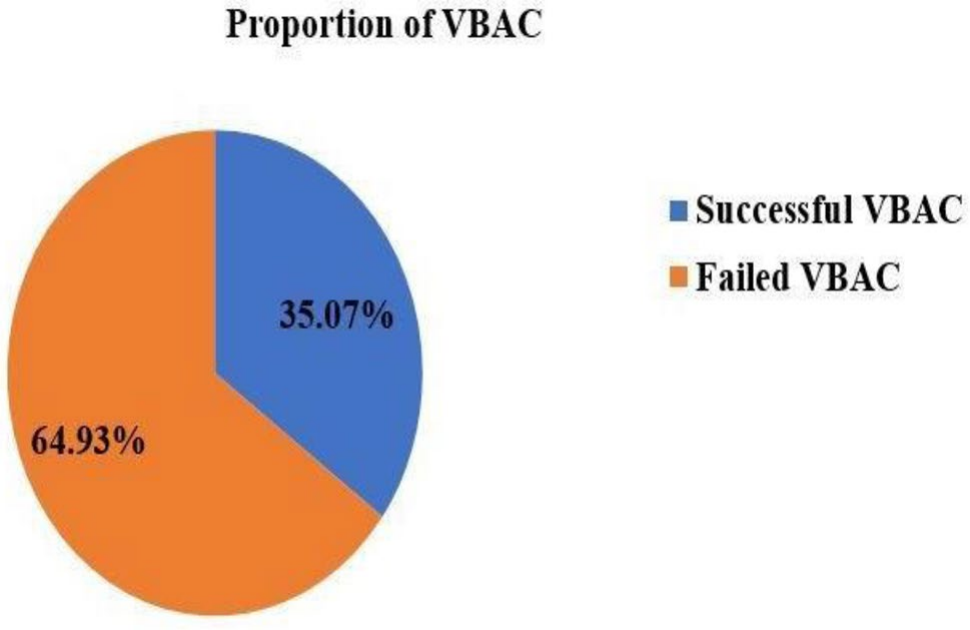
Proportion of successful VBAC in the public hospitals of Bahir Dar city, Northwest Ethiopia, 2021 (n = 345).

Out of 345 women who were included in the study, 121 (35.07%) [95% CI 29.9–40.0%] had successful VBAC (Fig. 2).

### Outcome of TOL

Among mothers who had successful VBAC, 88 (72.7%) gave birth spontaneously. Of the failed VBAC, NRFHRP (n = 87, 38.9%) and failure to progress (n = 72, 32.1%) were the main indications for emergency CS delivery (Table 2).

**Table 2:** Outcome of the trial of labor after cesarean section in the public hospitals at Bahir Dar City, Northwest, Ethiopia, 2021, (n = 345).

Variables	No. (%)
Mode of delivery	
Vaginal delivery	121 (35.1)
Emergency CS	224 (64.9)
Mode of vaginal delivery (n = 121)	
Spontaneous vaginal delivery	88 (72.7)
Instrumental delivery	33 (27.3)
Indication for emergency CS (n = 224)	
Failed progress of labor	72 (32.1)
NRFHRP	87 (38.9)
Uterine dysfunction	35 (15.6)
Others*	30 (13.4)
Timing of emergency CS (n = 224)	
The latent first stage of labor	78 (34.8)
The active first stage of labor	130 (58.0)
The second stage of labor	16 (7.2)

*Scar tenderness, presence of meconium, and cephalo-pelvic disproportion.

### Flamm and Geiger predictive model

**Figure 3:**
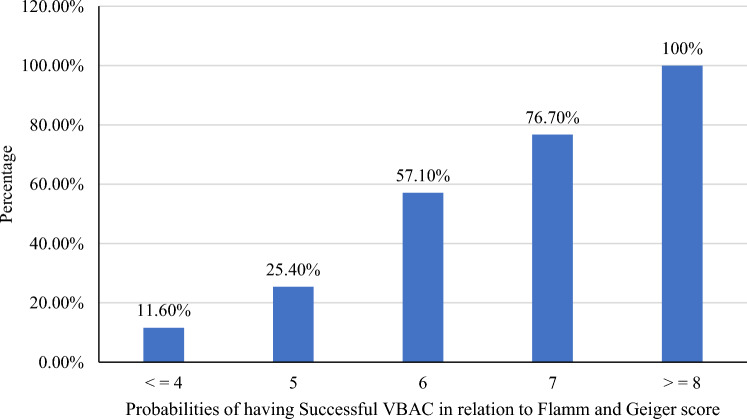
Successful VBAC rate according to Flamm and Geiger score value in the public hospitals of Bahir Dar city, Northwest Ethiopia, (n = 345).

Based on the Flamm and Geiger predictive model, the final cumulative VBAC score ranged from 1 to 10 in the present study. It was ≤ 3 in 22.0%, 4 in 27.8%, 5 in 17.1%, 6 in 14.2%, 7 in 8.7% and ≥ 8 in 10.2% of the cases. The mean score of successful VBAC was 7.11 ± 1.26 and for failed VBAC was 3.83 ± 0.94. The success rate of VBAC increased with increasing the total Flamm and Geiger score values: with the score of ≤ 4 the chance of successful VBAC was 11.6% and with a score of ≥ 8 it was almost 100% (Fig. 3).

**Figure 4:**
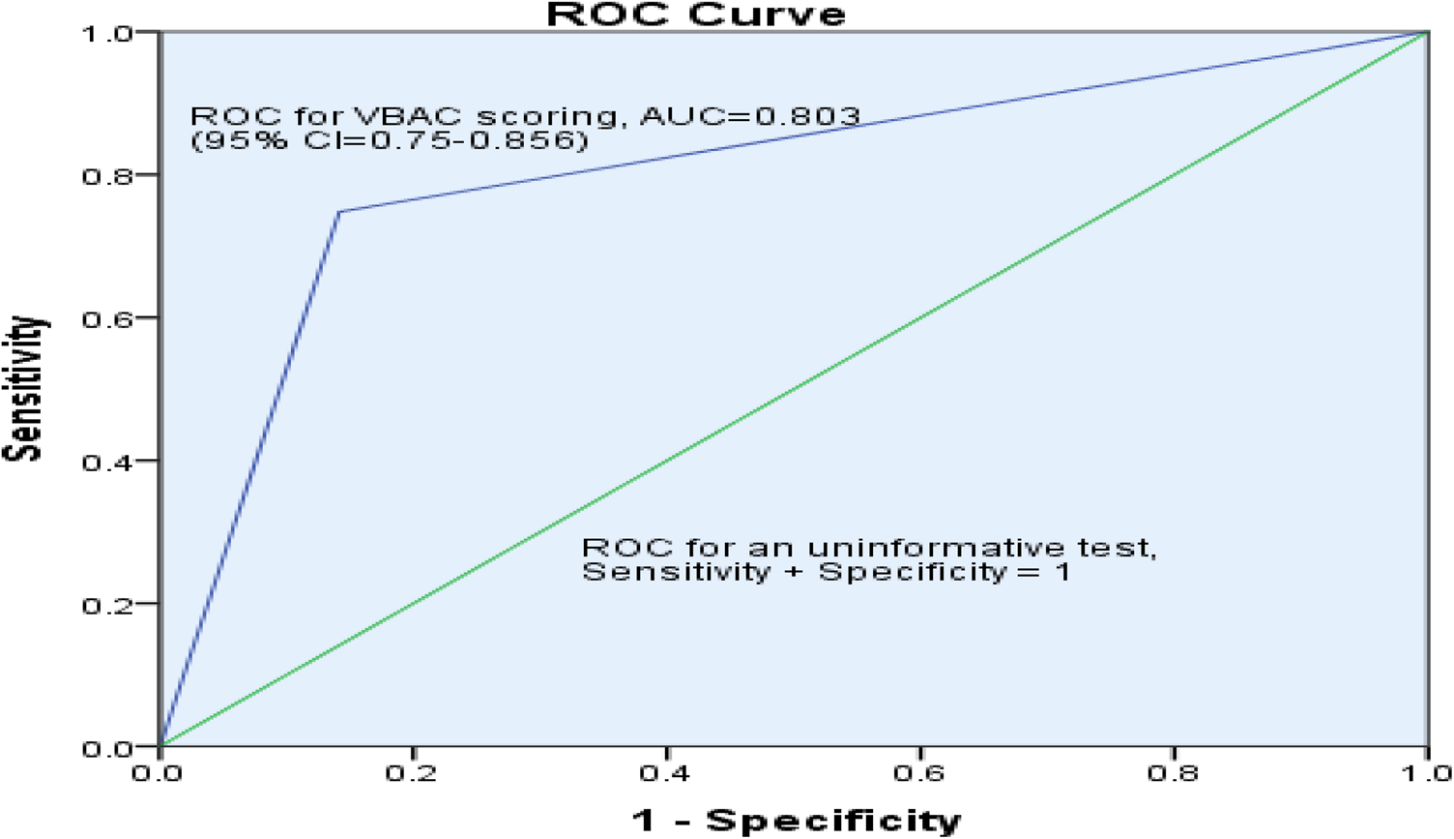
ROC curve for Flamm and Geiger predictive model in the public hospitals of Bahir Dar city, Northwest Ethiopia, (n = 345).

The ROC curve for VBAC scoring at a cut-off 5 score, the AUC is 0.803 (95% CI 0.75–0.856) with a P-value of < 0.001. The sensitivity and specificity were 73.6% and 86.6% respectively (Fig. 4).

**Figure 5:**
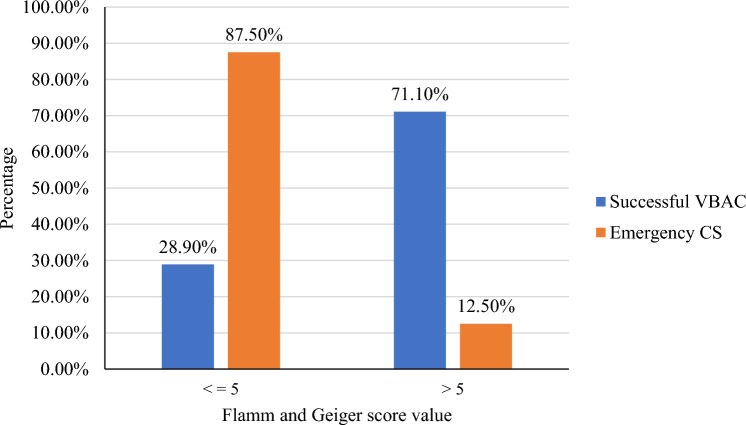
The relationship between the total Flamm and Geiger score with the TOLAC in the public hospitals of Bahir Dar city, Northwest Ethiopia (n = 345).

According to the Flamm and Geiger score, when the core was less than ≤ 5 the probability of repeated emergency CS was 87.50%, while when the score was > 5 the probability of successful VBAC was 71.10% (Fig. 5).

### Factors associated with successful VBAC

In binary logistic regression analysis; maternal age, address, parity, indication of primary CS, prior vaginal delivery, prior successful VBAC, amniotic membrane status, station of the presenting part, the position of the presenting part, cervical dilatation, and effacement on admission, gestational age, birth weight were candidate variables for multivariable analysis at a P-value of less than 0.25.

In a multivariable analysis mothers who are found in the age group of 21–30 years [AOR = 2.71, 95% CI 1.21–6.08], performing primary CS for non-recurring indications (NRFHRP and malpresentation) [AOR = 3.57, 95% CI 1.35–9.46] and [AOR = 4.21, 95% CI 1.59–11.13] respectively, women who had prior VBAC history [AOR = 9.46, 95% CI 3.55–25.16], previous vaginal delivery [AOR = 6.56, 95% CI 3.16–13.65], spontaneous rupture of membranes at admission [AOR = 4.18, 95% CI 2.01–8.71], cervical dilatation ≥ 4 cm [AOR = 2.20, 95% CI 1.06–4.56], cervical effacement ≥ 50% [AOR = 3.07, 95% CI 1.08–8.79], and low station (≥ 0 stations on pelvic examination) at admission [AOR = 2.77 95% CI 1.32–5.81] were significantly associated with successful VBAC After one LUTCS at a P-value of less than 0.05 (Table 3).

**Table 3:** Logistic regression analysis for successful VBAC in the public hospitals at Bahir Dar City, Bahir Dar, Northwestern Ethiopia, 2021, (n = 345).

Variables	VBAC		COR (95% CI)	AOR (95% CI)	P-value
	Successful	Failed			
Maternal age (years)					
31–42	18	70	1	1	
21–30	103	154	2.60 (1.46–4.62)	**2.71 (1.21–6.08)**	0.016*
Address					
Urban	65	155	1	1	
Rural	56	69	1.93 (1.23–3.05)	1.36 (0.70–2.64)	0.369
Parity					
I	30	133	1	1	
II	43	78	2.08 (1.20–3.59)	1.54 (0.70–3.42)	0.287
≥III	48	33	5.48 (3.01–9.97)	1.48 (0.53–4.13)	0.454
Reason for primary CS					
Failure to progress	21	63	1	1	
NRFHRP	28	29	2.90 (1.41–5.93)	**3.57 (1.35–9.46)**	0.011*
APH	10	34	0.88 (0.37–2.09)	0.64 (0.20–1.99)	0.436
Macrosomia	80	16	1.50 (0.56–4.01)	1.85 (0.45–7.59)	0.395
Malpresentation	37	32	3.47 (1.75–6.87)	**4.21 (1.59–11.13)**	0.004*
Unknown	6	16	1.12 (0.39–3.25)	1.97 (0.55–7.13)	0.300
Others**	11	34	0.97 (0.42–2.25)	1.02 (0.32–3.25)	0.970
Vaginal delivery before CS					
No	61	181	1	1	
Yes	60	43	4.14 (2.54–6.74)	**6.56 (3.16–13.65)**	0.001*
Vaginal delivery after CS					
No	78	212	1	1	
Yes	43	12	9.74 (4.88–19.43)	**9.46 (3.55–25.16)**	0.001*
Cervical dilatation					
< 4 cm	42	170		1	
≥4 cm	79	54	5.92 (3.65–9.60)	**2.20 (1.06–4.56)**	0.033*
Cervical effacement					
< 50%	6	59	1	1	
≥50%	115	165	6.85 (2.86–16.41)	**3.07 (1.08–8.79)**	0.036*
Gestational age (weeks)					
40–41^6/7^	64	160	1	1	
37–39^6/7^	24	97	1.62 (0.95–2.75)	1.45 (0.69–3.04)	0.330
Station					
High (< 0)	48	183	1	1	
Low (≥ 0)	73	41	6.79 (4.13–11.16)	**2.77 (1.32–5.81)**	0.007*
Position of presenting part					
OP/OT	17	67	1	1	
OA	104	157	2.61 (1.45–4.700)	1.23 (0.56–2.73)	0.604
Status of membrane					
Intact	60	191	1	1	
Ruptured	61	33	5.88 (3.52–9.83)	**4.18 (2.01–8.71)**	0.001*
Birth weight					
≥4000 g	6	21	1	1	
< 4000 g	115	203	1.98 (0.78–5.05)	1.81 (0.52–6.26)	0.348

Significant values are in [bold].

*P-value of < 0.05.

**Sever preeclampsia/eclampsia, IUGR, and Oligohydramnios.

## Discussion

In this study area, the TOLAC rate after one previous LUTCS was 69.5%, and the success rate of VBAC was 35.07% [95% CI 29.9–40.0%]. Based on Flamm and Geiger's predictive model, the mean score of successful VBAC was 7.11 ± 1.26 as against 3.83 ± 0.94 for failed VBAC. The successful rate of VBAC increased with increasing the total Flamm and Geiger score values: with the score of ≤ 4 the chance of successful VBAC was 11.6% and with a score of ≥ 8 it was almost 100%. The sensitivity and specificity were 73.6% and 86.6% respectively. Among mothers who had successful VBAC, 88 (72.7%) gave birth spontaneously, which is in line with a study conducted in India^51^. Of the failed VBAC, NRFHRP 87 (38.9%) and failed progress of labor, 72 (32.1%) were the main indications of emergency CS delivery. This finding is in line with a study done in Turkish^52^. More than half, (57.1%) of mothers gave birth within 8 h of the onset of labor with a mean of 9.4 ± 2.7 h for successful VBAC and 6.7 ± 2.9 h for failed TOLAC. The average age of the mothers who underwent VBAC was 26.50 ± 4.51 years and of those undergoing repeated emergency CS was 29.77 ± 4.52 years.

The success rate of VBAC in this study is almost in line with studies conducted at Mizan Tepi University teaching Hospital 41.0%^53^, Attat primary hospital in the Gurage zone (44.5%)^47^, South Africa 36%^54^, Nigeria 33.8%^55^, and St Stephens Hospital of New Delhi, India 40.0%^56^. However, the finding in this study is higher relative to studies done in Metu Karl referral hospital, southwest Ethiopia (24.7%)^57^, Iran (10.4%), Australia (14%), and India 24.2%^35,58,59^. The higher success rate of VBAC in this study might be due to the time gap between the years of the studies. As seen through time, the numbers of women who utilized maternal health care services increases and this could increase their chance of getting information about the risk and benefits of VBAC over repeated CS in the form of health education or counseling. There is supporting evidence from five years of reviews on VBAC that shows that adequate education and counseling are the cornerstones to having a high VBAC success rate with minimal adverse outcomes^60^.

The success rate of VBAC in this study is lower relative to a study conducted in teaching hospitals of Addis Ababa University (69.4%)^40^. The lower successful VBAC rate in this study could be a reflection of the increasing use of continuous electronic fetal monitoring, which helps to detect early things like non-reassuring fetal heart patterns. As seen in this study, the commonest indication for emergency CS in failed VBAC was fetal distress 87 (38.9%). Due to the pain of the labor, some women may change their minds to have repeated CS when things were not progressing as they expected. Additionally, women may not be prepared before or during pregnancy for their mode of delivery, may not be counseled during ANC visits on the advantage of VBAC over RCD, may not be encouraged during labor by health care providers or family members, having experienced long and difficult labor in a previous pregnancy, and lack of maternal psychological readiness for TOL. There is supporting evidence, which shows that preparing before or during on mode of delivery and getting counseling during ANC visits on the mode of delivery, as well as encouragement during labor, are important tools to manage TOLAC and increase the success of VBAC^6,61^.

The success rate of VBAC in this study is lower relative to studies conducted in different countries: like Nigeria, India, China, Thailand, Vietnam and Iraq shows that the success rate of VBAC was > 50%^33,52,60,62–73^. This discrepancy indicates the presence of differences in the utilization of maternal health care services, such as ANC and PNC services, which are the main entry points to counsel women after CS delivery on the mode of delivery in their future pregnancy. Furthermore, in Ethiopia, induction and augmentation are contraindicated and not practiced for a woman with previous CS^5^. Thus, if there is an insufficient uterine contraction in mothers who choose TOL, the only option is RCD. Most of the developed countries use epidural analgesia for a woman who tries labor after cesarean delivery, even for normal labor. Evidence from developed countries shows that the use of adequate pain relief such as epidural anesthesia helps encourage women to choose TOLAC and a have the high success rate of VBAC^61,74,75^. Additionally, the decision-making skills of the health care providers and the availability of necessary supportive material for the immediate management of potential complications affects the success rate of VBAC. Evidence shows that careful selection of women for a TOL will increase the success rate of VBAC and decrease the RCD rate^72,76^.

The maternal socio-demography and past and current obstetric-related factors were significantly associated with successful VBAC. Mothers who are found in the age group of 21–30 years were 2.71 times more likely to have successful VBAC relative to women who are found in the age group of 31–42 years. This is consistent with other studies^50,70,77^. Mothers who are found in the age group of less than 21–30 years may have few children and they may want to have more children in the future. This could make them psychologically ready for vaginal delivery. Women who had prior successful VBAC were 9.46 times more likely to have successful VBAC. The finding is in line with other studies^40,47,53,56,57,63,78–80^. Women who have previous success with VBAC may have good psychological readiness and they may also be aware of the advantage of vaginal delivery. Having prior VBAC indicates that the cause of primary CS is non-recurring, thus it may help the health care provider to avoid early judgment on the mode of delivery.

Women who had a vaginal delivery before CS were 6.56 times more likely to have successful VBAC. The finding is supported by other studies^8,40,50,53,55,56,62,65,81^. Women who have a history of prior vaginal delivery may have a better understanding of the advantage of vaginal delivery over CS. Having no prior vaginal delivery increases the risk of failed TOLAC^82^. Performing primary CS for non-recurring indications increases the chance of successful VBAC in subsequent childbirth; mothers who gave through CS for the indication of NRFHRP and malpresentation were 3.57 and 4.21 times more likely to have the chance of successful VBAC in their next childbirth. Mothers who had the non-recurring indication for primary CS could have a high chance of it not occurring in their next childbirth. This was supported by another finding^83^.

Admitting laboring women with a history of ruptured membranes increased the likelihood of successful VBAC by 4.18 times. It is congruent with other studies^5,8,40,79^. The possible reason might be that rapture of the membrane helps to release the natural prostaglandin and thus may in turn facilitate the progress of the labor. Similarly, women who were admitted with cervical dilatation of ≥ 4 cm were 2.20 times more likely to have successful VBAC. This finding is in line with other studies^47,53,60,65^. It may be due to the active first stage of labor, cervical dilation proceeding at its most rapid rate to complete cervical dilation (1.5 cm/h) relative to the latent phase. There is also supporting evidence from studies conducted in three teaching hospitals of Addis Ababa and India that showed, even if cervical dilation is greater than 3 cm at admission, it increases the favorability of successful VBAC^5,64^. The odds of having a successful VBAC were 3.07 times higher for women who were admitted with cervical effacement of ≥ 50%. It is in line with a study done in India^51^. This may be attributed to having effaced and thin cervical status may indicate the favorable progress of the labor, which in turn increases the success of VBAC in TOLAC**.**

Women who were admitted with a low station (≥ 0 stations on pelvic examination) were 2.77 times more likely to have successful VBAC. This finding is consistent with a study done in the teaching hospitals of Addis Ababa University^40^. The low station of the presenting part indicates that the women are more likely to have a favorable bishop score and this may make them have a successful VBAC. There is a supporting report from a study conducted in St Stephens Hospital of New Delhi, India shows that women who had favorable bishop scores on admission were more likely to have successful VBAC^56^.

### Strengths and limitations of the study

In our study, we tried to assess the external validation of the Flamm and Geiger predictive model for mothers who had prior LUTCS. This predictive model is important to counsel women who initially opt for TOL, and change their minds after the onset of labor to assure that they have a high likelihood of successful VBAC. This study has certain limitations. Since it was a retrospective study, maternal and fetal information was abstracted from the mother chart, and some variables like maternal height, weight, marital status, occupation, and complete components of the bishop’s score were not included. We also found some women charts that had no complete maternal or fetal information. As a solution, the incomplete charts were replaced by complete women charts. The close-to-delivery nomogram or algorithm recommended by Grobman (Maternal–Fetal Medicine Unit calculator) was not assessed because of the nature of the study design, it was impossible to get the complete components of the bishop’s score and the maternal body mass index. The Grobman model is valid for use before the onset of labor when evidence for counseling is critical.

## Conclusion

In this study area, the TOLAC rate is encouraging, however, the success of VBAC was lower relative to the majority of studies conducted in different countries. Women who are found in the age group of 21–30 years, who gave birth by CS for the indication of non-requiring cases in primary CS, women who have a history of vaginal delivery before or after the primary CS, and women who are presented with cervical dilatation of ≥ 4 cm, cervical effacement ≥ 50%, low station (≥ 0 stations on pelvic examination) and ruptured membrane have a high chance of successful VBAC. When a woman has a CS before she leaves the hospital, she should know why the CS was performed, the type of incision that was performed, and what impact the CS will have on her subsequent childbirth. Counseling should be strengthened during the ANC visits and women should be fully involved in the decision-making process about the mode of delivery. In our study, based on the predictive model, we found that careful selection of mothers for TOLAC increases the success rate of VBAC. The successful rate of VBAC increases with increasing the total Flamm and Geiger score values. Finally, we emphasize a need to develop a well-defined management protocol to increase the number of successful VBACs and bring down the overall CS rate.

## Data Availability

All related data has been presented within the manuscript. The data set supporting the conclusion of this article is available from the corresponding author upon reasonable request (Wondu Feyisa Balcha (wondufeyisaa85@gmail.com).

## Abbreviations

ANC: Antenatal care
AOR: Adjacent odd ratio
AUC: Area under curve
CI: Confidence interval
COR: Crude odd ratio
CS: Cesarean section
LUTCS: Lower uterine transverse cesarean section
RCD: Repeated cesarean delivery
RCO: Receiver operating characteristic
TOL: Trial of labor
TOLAC: Trial of labor after cesarean
VBAC: Vaginal birth after cesarean section
WHO: World Health Organization
